# Mesoscopic Damage Characteristics of NEPE Propellant Under Drop-Weight Impact

**DOI:** 10.3390/ma19091773

**Published:** 2026-04-27

**Authors:** Zhibo Zhang, Zhensheng Sun, Yuxiang Liu, Yujie Zhu, Yu Hu

**Affiliations:** Zhijian Laboratory, Rocket Force University of Engineering, Xi’an 710025, China; 18842349865@163.com (Z.Z.); liuyx@smail.nju.edu.cn (Y.L.); yujiezhu92@163.com (Y.Z.); huyu1222@163.com (Y.H.)

**Keywords:** NEPE propellant, drop-weight impact, micro-CT, SPH-FEM coupling algorithm, mesoscopic damage law

## Abstract

During the production, storage, and use of solid rocket motors, the impact generated by unexpected accidents, such as collision or drop, will cause damage to the propellant and affect the safety of the motor. However, the progressive evolution mechanism of mesoscopic damage in NEPE propellant under such impact conditions has not been fully elucidated, and there is still a lack of quantitative method to evaluate the impact-induced damage degree, which restricts the engineering safety assessment of solid rocket motors. To investigate the influence mechanism, the mesoscale damage characteristics of NEPE propellant under drop-weight impact is systematically studied. First, damaged NEPE specimens are obtained by conducting drop-weight experiments with a 10 kg hammer, where the drop height is varied to apply different impact impulses. The internal meso-structure of the propellant is then characterized using micro-CT, yielding detailed imagery of the refined meso-structural features and damage morphologies in the NEPE propellant. To capture the dynamic evolution process of mesoscale damage, a mesoscopic model incorporating AP, Al, HMX particles and voids, is subsequently constructed based on the high-precision mesoscopic morphology characterized by micro-CT. By integrating the deviatoric constitutive model, Gurson plastic damage model, and bilinear cohesive zone model, high-fidelity numerical simulations of the drop-weight impact damage process are performed using the advanced SPH-FEM coupling algorithm. The results indicate that no significant damage occurs when the impact impulse is less than 13.85 N·s. As the impulse increases, phenomena including matrix microcracks, void collapse, particle/matrix interface debonding, and main crack formation appear sequentially. When the impulse exceeds 24.25 N·s, particle fragmentation and transgranular fracture occur, accompanied by plastic flow and frictional heating that induce ignition. Finally, the overall damage degree is fitted by the Boltzmann function, and a function for quantitatively describing the damage degree is obtained, which can provide theoretical support for the impact safety assessment of solid rocket motors.

## 1. Introduction

NEPE propellant is a composite energetic material containing a high proportion of solid particles. It is widely used in solid rocket motors due to its high energy density, high specific impulse, and excellent low-temperature mechanical properties. Solid rocket motors may be subjected to accidental mechanical impacts, vibrations, and shaking during production, storage, or transportation, potentially leading to unexpected incidents such as detonation or deflagration. Under dynamic loading, various types of mesoscopic damage, including micro-cracking, interfacial friction, void collapse, and viscous shear, may develop in particulate-filled NEPE propellant [1]. The accumulation of such damage can lead to alterations in the macroscopic mechanical properties of the propellant, potentially resulting in critical issues such as fracture and the formation of localized hot spots, which severely compromise the safety of solid rocket motors. Therefore, investigating the meso-damage in NEPE propellant following impact loading is of paramount importance for the safety assessment and risk control of solid rocket motors in engineering applications.

Current research on the impact safety of NEPE propellant primarily employs experimental and numerical simulation approaches [2,3]. For impact experimental methods, existing studies have carried out extensive investigations on the dynamic mechanical response, failure and ignition mechanism of NEPE propellant under different loading conditions. Sun et al. [4] conducted high-strain rate (1500–4500 s^−1^) impact tests on NEPE propellant using SHPB, and revealed that transgranular fracture, matrix tearing, and void coalescence are the dominant failure modes under high-strain rate loading. However, this work focused on high-strain rate conditions, and did not involve the mesoscopic damage evolution of NEPE propellant under low-speed drop-weight impact. Guo et al. [5] further conducted SHPB experiments at various temperatures and high-strain rates, and found that frictional shear heating among AP particles is the main ignition trigger. This work also focused on strong impact scenarios, and did not clarify the critical impulse threshold of mesoscopic damage initiation under low-speed drop impact. Li et al. [6] investigated the effect of confining pressure on the compressive mechanical properties of NEPE propellant, and established a pressure-dependent nonlinear constitutive model, but did not involve the impact damage characteristics of the propellant. Hu et al. [7] optimized the bilinear cohesive zone model parameters of NEPE propellant through quasi-static tensile tests, and analyzed the mesoscopic damage evolution under quasi-static loading, while the dynamic damage evolution under impact loading was not involved. Xing et al. [8] conducted in situ SR-CT tensile tests on NEPE propellant under finite deformation, and observed the mesostructural evolution under quasi-static tensile loading, but the dynamic meso-damage mechanism under drop-weight impact was not systematically revealed. For low-speed drop-weight impact, which corresponds to the accidental drop scenario of solid rocket motors, existing studies [3,9,10] have investigated the hot-spot formation and ignition mechanism of composite solid propellants and explosives under low-amplitude, long-pulse impact, while the mesoscopic damage evolution mechanism of NEPE propellant under such loading conditions has not been systematically revealed.

For the mesoscopic damage characterization of composite propellants, optical microscopy (OM), scanning electron microscopy (SEM), ultrasonic testing (UT), and micro-computed tomography (micro-CT) are the most widely adopted methods [11]. OM [12] and SEM [13] are capable of acquiring high-resolution meso-morphological features of solid propellants, and have been widely used to observe the surface damage morphology of NEPE propellant after dynamic loading [4,5]. However, both techniques are limited to characterizing the meso-structure at the propellant’s surface and cannot probe the internal three-dimensional meso-structure. UT offers advantages in real-time inspection and quantitative damage characterization, but it struggles to accurately resolve the specific locations of mesoscopic damage or capture the morphological changes in damage evolution due to the complexity of the propellant’s meso-structure. In contrast, micro-CT technology can non-destructively detect the internal structural details of propellants with high precision, and realize quantitative characterization of internal mesoscopic damage [14]. Collins [15] and Pei [16] reconstructed the three-dimensional morphology of HTPB solid propellant using micro-CT, and established a representative volume element (RVE) model based on the real meso-structure. Li et al. [17] reconstructed meso-structural models of the internal components and interfaces within HTPB propellant utilizing micro-CT scanning, thereby analyzing the dewetting evolution process. Liu et al. [18] conducted uniaxial tensile in situ CT tests, capturing the evolution of the internal mean gray value and average porosity with tensile strain, alongside corresponding CT images and attenuation coefficient curves. Ma et al. [19] employed micro-CT to perform a scanning analysis of HTPB propellant, obtaining 2D tomographic images and revealing the 3D spatial distribution of its particulate constituents. However, there is still a lack of micro-CT based non-destructive characterization of the internal three-dimensional meso-damage evolution of NEPE propellant under drop-weight impact.

Regarding numerical simulation, the cohesive zone model (CZM) is commonly used in numerical simulations for characterizing damage in composite materials, with the bilinear [20] and exponential [21] models being among the most widely adopted. Barenblatt proposed the formulation of different fracture criteria based on the specific mechanical properties of the cohesive material when calculating material fracture. In studying interfacial dehumidification, CZM assume the interface consists of a single layer of elements, where the forces and deformation displacements acting on these elements are defined through various modeling approaches. Once the traction force reaches the maximum strength that the interfacial element can sustain, damage initiation occurs. This is followed by a degradation of the load-bearing capacity. The element fails completely when the separation displacement reaches the critical failure displacement. This fundamental relationship governing the damage process is referred to as the traction-separation law. Hou et al. [22] conducted experimental and numerical investigations on the meso-damage of HMX-MDB propellant under tensile loading using SEM. The study verified that a segmented CZM provides a more accurate description of the damage evolution. It was further demonstrated that the “dewetting-nucleation-crack propagation” process is predominantly governed by the relative strengths of the interface and the binder matrix. Francqueville et al. [23] employed a micromechanical approach, integrating experimental data and finite element analysis, to establish the relationship between microstructural evolution and macroscopic mechanical properties in solid propellants. The study further revealed that cohesive parameters significantly influence both the initiation and propagation of local damage, as well as the material’s macroscopic stress–strain response. Ma et al. [24] conducted numerical simulations of interfacial Type I and Type II damage using a PPR CZM that accounts for different tangential and normal fracture energies, concluding that this model is more suitable for mixed damage than the bilinear CZM. Currently, the FEM serves as the predominant numerical simulation technique employed in the study of composite energetic materials. The SPH-FEM coupling algorithm integrates the maturity of FEM in structured mesh processing with the flexibility of SPH [25] in handling large deformations and fracture phenomena, making it applicable for damage evolution calculations in composite energetic materials. Johnson [26] applied the SPH-FEM coupling algorithm to various impact dynamics problems and successfully integrated this method into the impact dynamics software EPIC (version 2002, Alliant Techsystems Inc., Hopkins, MN, USA). However, there is still a lack of high-fidelity SPH-FEM coupling numerical model that can accurately reproduce the whole process of multi-mode mesoscopic damage coupling evolution of NEPE propellant under drop-weight impact.

It can be observed that the meso-structure critically influences both the macroscopic properties of solid propellants and the safety of rocket motors. Existing studies have carried out extensive investigations on the dynamic mechanical properties, failure and ignition response of NEPE propellants under high-strain rate loading via SHPB experiments, revealing the influence of strain rate and temperature on the macroscopic mechanical response and microscopic failure modes of NEPE propellants. However, these studies mainly focus on the high-strain rate conditions corresponding to strong impact and explosion scenarios, while the meso-damage mechanism of NEPE propellants under low-speed drop-weight impact, which is more consistent with the accidental drop/collision scenario in actual engineering, has not been systematically revealed. Meanwhile, existing studies mostly adopt SEM and OM to observe the surface damage morphology of specimens, and there is still a lack of non-destructive characterization of the internal three-dimensional meso-damage evolution inside NEPE propellants under impact loading. To date, research on the mesoscopic damage mechanism of NEPE propellant under low-speed drop-weight impact is still insufficient, especially the quantitative relationship between impact impulse and mesoscopic damage evolution has not been clearly revealed. Specifically, the critical impact impulse thresholds for the initiation and evolution of different mesoscopic damage modes (including matrix microcracks, void collapse, particle/matrix interface debonding, main crack formation, particle fragmentation and transgranular fracture) in NEPE propellant have not been quantitatively clarified, the progressive evolution law of multiple meso-damage mechanisms under increasing impact impulse has not been fully elucidated, and there is no quantitative function for describing the overall damage degree of NEPE propellant under different impact impulses, which cannot provide effective theoretical support for the impact safety assessment of solid rocket motors.

To address this issue, this work studies the meso-damage characteristics of NEPE propellant under drop-weight impact conditions through experiments and numerical simulations. In this study, the drop-weight impact experiments are used to simulate the process of the solid rocket motor being subjected to external impacts. The impacted NEPE propellant is characterized using micro-CT scanning, and image reconstruction yields high-resolution visuals that directly reveal its meso-structural features and damage morphology. Based on these image characteristics, a mesoscopic model incorporating AP, Al, HMX particles and voids is constructed based on the high-precision mesoscopic morphology characterized by micro-CT. By integrating the deviatoric constitutive model, Gurson plastic damage model, and bilinear cohesive zone model, high-fidelity numerical simulations of the drop-weight impact damage process are performed using the advanced SPH-FEM coupling algorithm. The simulation results elucidate the characteristic modes and evolution patterns of multiple meso-damage mechanisms within the propellant. Furthermore, the analysis quantifies the damage effects induced by drop-weight impact on NEPE propellant, leading to the establishment of an “impact impulse–meso-damage” model, and a function for quantitatively describing the damage degree is obtained via fitting by the Boltzmann function. The remainder of this paper is organized as follows.

In Section 2, the experiment of drop-weight impact is carried out. Section 3 gives the SPH-FEM coupling algorithm. Section 4 presents the results of numerical simulation. Conclusions are summarized in Section 5.

## 2. Drop-Weight Impact Experiment

This section aims to obtain the mesoscopic damage morphology of NEPE propellant under different impact impulses through drop-weight impact experiment and micro-CT characterization, clarify the critical impulse threshold of damage initiation and ignition, and provide experimental basis, model parameters and verification benchmark for the subsequent numerical simulation and quantitative damage model establishment.

The drop-weight impact method is selected based on the actual loading scenario. During the production, storage, transportation and use of solid rocket motors, the accidental impact caused by collision or drop is low-speed and low-strain-rate impact, which can be accurately simulated by drop-weight impact. The SHPB method is mainly used for high-strain-rate loading conditions such as strong impact and explosion, which is not consistent with the accidental impact scenario concerned in this work. In addition, the drop-weight impact can apply stable and controllable impact impulses, and cooperate with micro-CT to realize the non-destructive characterization of internal meso-structure and damage morphology of NEPE propellant, which meets the research requirements of mesoscopic damage characteristics and evolution law under real accidental impact. The NEPE propellant, whose formulation is primarily composed of binders, oxidizers, explosives, and fuels [27], is first cut into specimens of Ø(5–10) mm × (2–6) mm using cutting tools and molds.

### 2.1. Experimental Apparatus and Method

Micro-CT scanning enables non-destructive observation of internal structures while providing quantitative analysis of damage. However, current research primarily focuses on using micro-CT to characterize processes such as tensile and shear testing in composite materials. This study employs a Skyscan 1172 micro-CT apparatus to characterize the meso-damage in NEPE propellant induced by drop-weight impact. This apparatus is a multi-range nanoscale 3D X-ray micro-CT system featuring a unique X-ray source and detector geometry. Its core components include a microfocus X-ray tube with a high-voltage power supply, a precisely controllable sample stage, a 2D X-ray CCD camera, and an electronic computer equipped with dual processors and an LCD monitor. Through its precise spiral reconstruction technique, the system effectively eliminates cone-beam CT artifacts, thereby generating clearer, high-precision images. During operation, the specimen stage is pre-set to rotate 360° at specific angular increments. As the stage rotates through each incremental step, the system acquires a set of processed projection images following X-ray exposure. These sequential X-ray projections collectively constitute a complete dataset for tomographic reconstruction.

**Figure 1:**
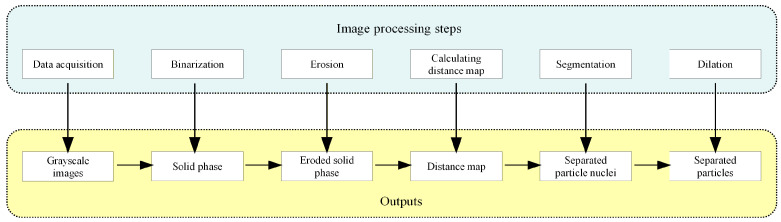
Image reconstruction flowchart.

The projection data is processed using a corresponding reconstruction algorithm to generate cross-sectional data of the specimen. By sequentially stacking these cross-sectional slices, a series of 2D grayscale tomographic images is ultimately produced [28]. The complete reconstruction flowchart is illustrated in Figure 1. The specimens are scanned using the micro-CT system to obtain their meso-morphology prior to impact for each specimen group.

**Figure 2:**
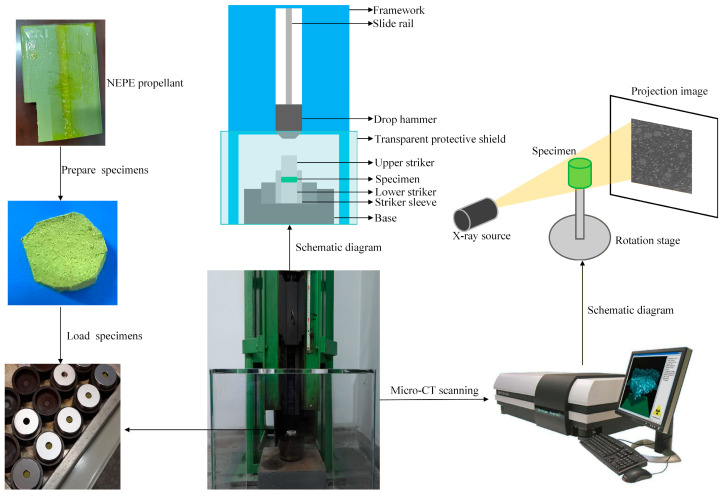
Experimental systems.

To simulate the effect of external impact on the meso-structure of NEPE propellant in solid rocket motors, a drop-weight experiment apparatus is employed. The specimen is placed within an anvil assembly, constrained by upper and lower impact platens. By varying the mass and height of the drop hammer, impact loads are applied to NEPE propellant specimens of different dimensions. The impacted specimens are then scanned again using the micro-CT system for image reconstruction, enabling analysis of how external impact influences the meso-structure and induces various damage modes in the propellant. The experimental system is illustrated in Figure 2.

### 2.2. Experimental Results and Discussion

To determine the range of drop heights that induce meso-damage without causing a macro-response, impact sensitivity experiments are conducted. The experiment parameters are shown in Table 1.

**Figure 3:**
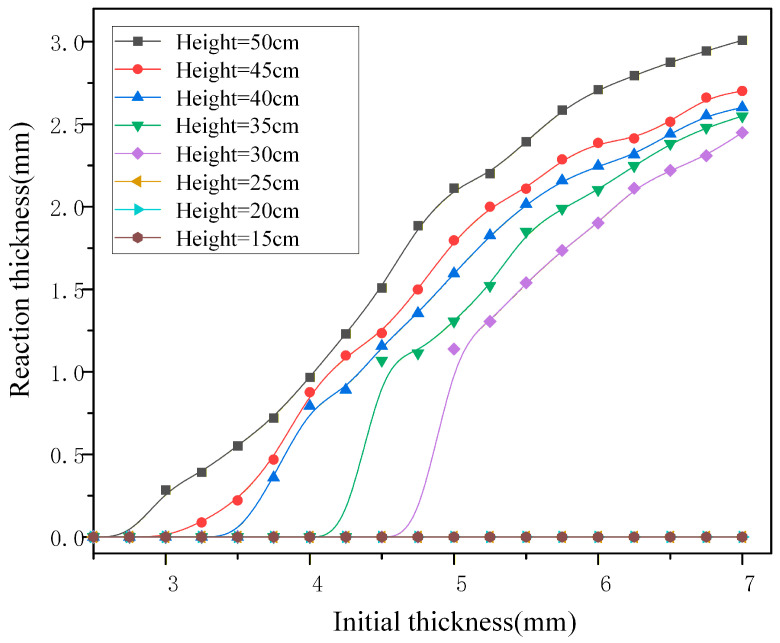
Reaction thickness–specimen thickness curve.

The specimens are divided into ten groups with varying thicknesses. For each group, three replicate experiments are conducted at different drop heights, resulting in a total of 240 experiments. The curve fitted from the data, illustrating the relationship between reaction thickness and specimen thickness, is shown in Figure 3.

Experimental results reveal that the ignition response of the NEPE propellant formulation used in this study is influenced not only by the impact impulse but also by the initial thickness of the specimens. Based on 240 experiments, ignition occurs only when a 10 kg drop hammer is released from a height of 50 cm, and solely for specimens with thicknesses below 3 mm. As the specimen thickness increases, the impact ignition threshold gradually decreases. When the thickness exceeds 5 mm, the impact ignition threshold in the drop-weight experiment stabilizes at 24.25 N·s. The measurement of this ignition threshold is only used to determine the upper limit of impact impulse for the subsequent non-ignition mesoscopic damage experiments, to ensure that the whole process of damage evolution before ignition can be systematically studied. The meso-damage characteristics and density in the most severely damaged subsurface region remain essentially consistent across different thicknesses. This indicates that the experimental results are not affected by the specimen size in the conventional size range of engineering propellant charges, and have good generalizability for the actual accidental drop impact scenario of solid rocket motors.

For small NEPE propellant specimens, ignition does not lead to complete consumption of the material. Experimental observations indicate that the reaction is primarily confined to the upper portion of the specimen, while the lower section remains largely unreacted. The mechanical wave generated by drop-weight impact undergoes progressive attenuation as it propagates through the propellant. According to Reference [3], the dominant ignition mechanism is identified as the formation of localized hot spots due to plastic flow and friction within the propellant matrix. With increasing impact impulse, the region experiencing the highest mechanical wave intensity within the propellant, where plastic flow is most severe, becomes more prone to localized heating and hotspot formation, consequently exhibiting a higher propensity for ignition. The reaction typically encompasses approximately 10% to 45% of the total specimen thickness.

To investigate whether the safety of the solid rocket motor is affected when it is subjected to external dynamic loads without ignition and explosion, the specimens analyzed in this study are specifically selected from those that do not ignite upon impact. By comparing micro-CT reconstructed images of the specimens before and after impact, distinct meso-structural damage is identified in the NEPE propellant subjected to external loading. The predominant failure mechanisms are characterized as matrix tearing and interfacial dewetting between the particles and the matrix. As indicated in Reference [29], transgranular fracture and particle fragmentation only occur when a 10 kg drop hammer reaches a height of 30 cm. However, since the propellant specimens in our experiments had already exhibited ignition responses under such conditions, which do not meet the specimen selection criteria, the impact height is maintained below 30 cm. Consequently, no transgranular fracture or particle fragmentation is observed in the scanning reconstruction results. Due to their dispersed distribution and sub-resolution size, matrix voids cannot be directly resolved in the micro-CT results. Therefore, the influence of impact loading on the voids within the NEPE propellant is analyzed by calculating the porosity from CT slice data before and after experiment. This data-driven approach subsequently enables the construction of a three-dimensional void model.

**Figure 4:**
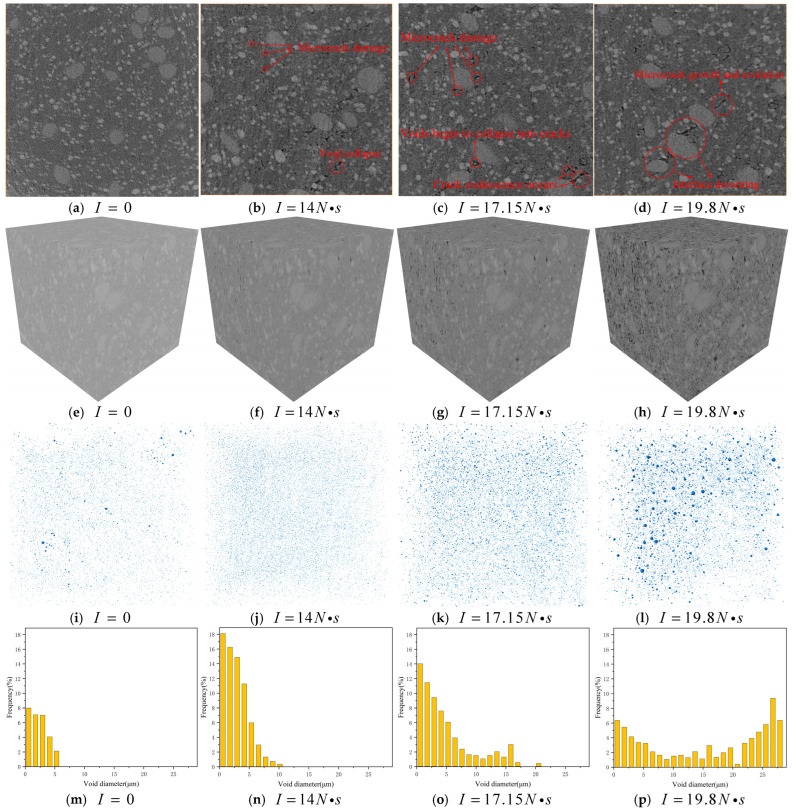
(**a**–**d**) Two-dimensional cross-sectional slices of specimen damage, (**e**–**h**) three-dimensional reconstruction models of specimen damage, (**i**–**l**) void distribution, and (**m**–**p**) calculation results of porosity under impacts with different impulses.

Owing to the pronounced viscoelastic characteristics of NEPE propellant under real conditions, the intensity of mechanical waves attenuates progressively with increasing penetration depth. Consequently, the most severe meso-damage is generated in the surface region of the propellant, which poses the greatest potential hazard to the safety of solid rocket motors [9]. Therefore, the specimens with a side length of 2 mm are cut at a depth of 1 mm in the central area of the surface for scanning characterization. For the 2 mm edge-length specimens, the results under different impact impulses, including 2D cross-sectional slices, 3D reconstructed models, void distribution maps, and calculated porosity values are presented in Figure 4.

In Figure 4i–l, the spherical blue scatter points represent the equivalent volume substitution of voids or micro-cracks within the binder matrix, which can be used to calculate the number and equivalent volume of damage. Analysis of specimen porosity and micro-CT reconstructed images reveals that when the specimen thickness exceeds 3 mm, the meso-damage characteristics and density in the most severely damaged subsurface region remain essentially consistent across different thicknesses. During the manufacturing process of NEPE propellant, voids ranging from 0 to 10 μm in size are formed. When the impact impulse reaches 13.9 N·s, micro-cracks approximately 5–10 μm in size are first observed, exhibiting a scattered, point-like distribution. This phenomenon is attributed to the heterogeneous distribution of particles during the manufacturing of NEPE propellant, which generates localized low-density regions. These regions are particularly susceptible to initial damage via viscous shear when subjected to mechanical stress waves. When the impact impulse exceeds 14 N·s, initial voids begin to collapse, accompanied by the propagation of micro-cracks. Due to the reflection and superposition of mechanical waves at particle/matrix interfaces, wave intensity becomes amplified, inducing misalignment tendencies between particles. Consequently, the regions adjacent to interfaces become more prone to micro-crack damage, with new micro-cracks predominantly forming around particles. When the impact impulse exceeds 17.15 N·s, the proportion of initial voids decreases significantly. Subjected to longitudinal impact loading, these voids progressively collapse and evolve into cracks extending tangentially to the direction of mechanical wave propagation. Concurrently, micro-cracks continue to grow and coalesce. When the impact impulse exceeds 19.8 N·s, localized dewetting occurs at the particle/matrix interfaces, while initial voids collapse into transverse cracks. Concurrently, propagating micro-cracks coalesce to form larger macro-cracks. When the impact impulse exceeds 22.14 N·s, interfacial dewetting damage evolves into large-scale crack damage. The coalescence of numerous propagating cracks forms dominant macro-cracks, leading to visibly observable matrix tearing damage in the NEPE propellant at the macroscopic scale. Consequently, the mechanical properties of the propellant undergo significant degradation, and it progressively loses its load-bearing capacity. When the impact impulse exceeds 24.25 N·s, transgranular fracture and fragmentation of solid particles occur [29], with intensified plastic flow and friction generating hot spots that ultimately lead to specimen ignition.

## 3. SPH-FEM Coupling Algorithm

This section introduces the SPH-FEM coupling algorithm adopted in this work. The above experimental results clarify the progressive evolution law of mesoscopic damage of NEPE propellant with the increase in impact impulse, and determine the critical impulse threshold of each damage stage. However, the experiment can only obtain the static damage morphology before and after impact, and cannot reveal the dynamic evolution process of mesoscopic damage in the impact process. The core purpose of selecting this algorithm is to accurately simulate the dynamic damage evolution process.

Since 1994, the SPH-FEM coupling algorithm has been utilized in pioneering work for numerical simulations of complex systems [30,31]. This algorithm employs the SPH method for calculating regions with large deformations, fractures, and fragmentation, while utilizing the FEM method in areas with relatively minor deformations. This approach achieves a balance between computational efficiency and simulation accuracy. However, current research primarily focuses on applying the SPH-FEM coupling algorithm to simulate macroscopic phenomena. To model the meso-damage and failure processes of NEPE propellant under impact loading, this study employs the SPH-FEM coupling numerical approach. Simultaneously, a bilinear CZM is introduced within the FEM domain to accurately characterize interfacial damage initiation and evolution.

The algorithm employs a second-order accurate leapfrog method for the numerical integration of the SPH equations. In this scheme, particle density, velocity, and energy are updated using Δt/2 step, while coordinates are updated using Δt step. Subsequently, after the coordinate update, the particle density, velocity, and energy are advanced by another Δt/2 step, thereby synchronizing their time levels with the particle coordinates. To enhance computational efficiency, a linked list search method [32] is employed for nearest neighbor particle search. Before each time step, the temporary linked list settings are updated, assigning particles to their respective linked lists to search for neighboring particles. The fully variable smoothing length SPH method [33,34,35] is employed, coupled with an artificial stress formulation [36], to enhance computational efficiency while simultaneously improving numerical accuracy and mitigating tensile instability.

**Figure 5:**
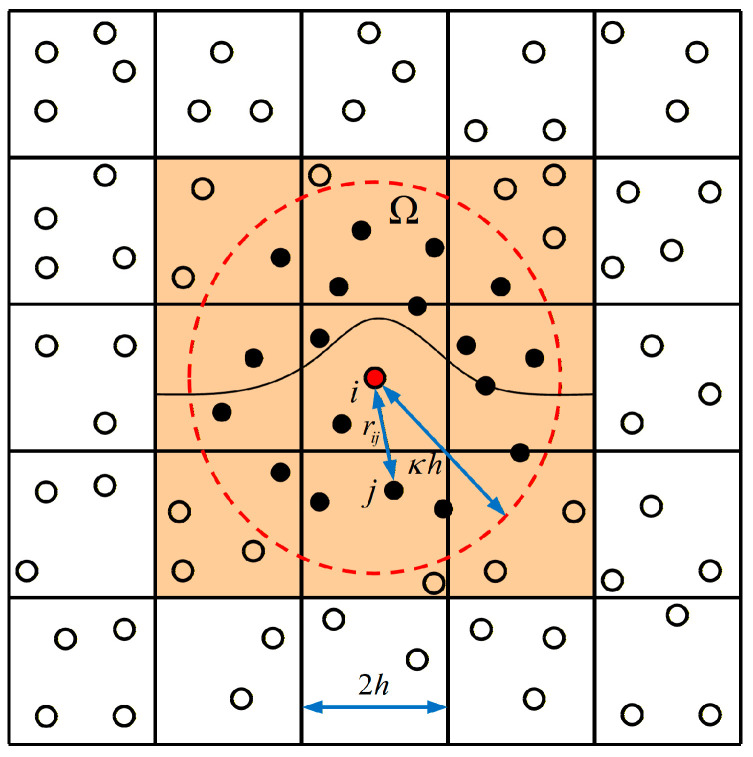
Schematic of the cell-linked list method for neighbor search.

Figure 5 shows how to search for cells using the linked list method.

This work employs a lumped mass matrix approach, which concentrates the elemental mass at the nodal points, to facilitate the coupling between SPH particles and finite elements.

(1) $${\left(M_{l}^{e}\right)}_{ij}=\left\{\begin{array}{cc} \sum_{k=1}^{n_{e}}{\left(M^{e}\right)}_{ik}=\sum_{k=1}^{n_{e}}\int_{V_{e}}\rho N_{i}^{T}N_{k}dV & \left(j=i\right)\\ 0 & \left(j\neq i\right) \end{array}\right.$$

where $n_{e}$ is the number of nodes; ρ is density; e is energy; and M is the mass matrix.

**Figure 6:**
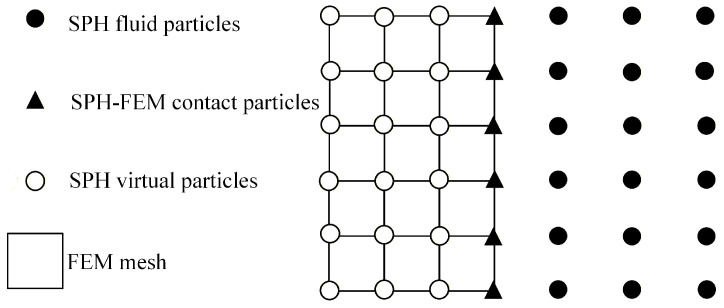
Coupling forms of particles and nodes.

The configuration of the coupling between SPH particles and finite element nodes at the interface is illustrated in Figure 6.

The information transfer at the coupling interface is achieved through the smoothing kernel function W and the shape function N. Specifically, the physical quantities of SPH particles at the interface are exchanged with FEM nodes employing the following formulation:

(2) $$\frac{d\rho_{i}}{dt}=\sum_{j=1}^{N}m_{j}(v_{i}^{\beta }-v_{j}^{\beta })\frac{\partial W_{ij}}{\partial x_{i}^{\beta }}+\sum_{j=1}^{N_{b}}m_{bj}(v_{i}^{\beta }-v_{bj}^{\beta })\frac{\partial W_{ij}}{\partial x_{i}^{\beta }}$$

(3) $$\frac{dv_{i}^{\alpha }}{dt}=\sum_{j=1}^{N}m_{j}(\frac{\sigma_{i}^{\alpha \beta }}{\rho_{i}^{2}}+\frac{\sigma_{j}^{\alpha \beta }}{\rho_{j}^{2}})\frac{\partial W_{ij}}{\partial x_{i}^{\beta }}+\sum_{j=1}^{N_{b}}m_{bj}(\frac{\sigma_{i}^{\alpha \beta }}{\rho_{i}^{2}}+\frac{\sigma_{bj}^{\alpha \beta }}{\rho_{bj}^{2}})\frac{\partial W_{ij}}{\partial x_{i}^{\beta }}$$

(4) $$\frac{de_{i}}{dt}=\frac{1}{2}\sum_{j=1}^{N}m_{j}(v_{j}^{\beta }-v_{i}^{\beta })(\frac{\sigma_{i}^{\alpha \beta }}{\rho_{i}^{2}}+\frac{\sigma_{j}^{\alpha \beta }}{\rho_{j}^{2}})\frac{\partial W_{ij}}{\partial x_{i}^{\beta }}+\frac{1}{2}\sum_{j=1}^{N_{b}}m_{bj}(v_{bj}^{\beta }-v_{i}^{\beta })(\frac{\sigma_{i}^{\alpha \beta }}{\rho_{i}^{2}}+\frac{\sigma_{bj}^{\alpha \beta }}{\rho_{bj}^{2}})\frac{\partial W_{ij}}{\partial x_{i}^{\beta }}$$

(5) $$\frac{dx^{\alpha }}{dt}=v^{\alpha }$$

(6) $$\langle f(x)\rangle =\sum_{i}N_{i}(x)f(x_{i})$$

where $m_{bj}$ is the mass of the background particle j; $\rho_{bj}$ is its density; $v_{bj}$ is the velocity vector; $\sigma_{bj}$ is the stress tensor; $N_{i}(x)$ is the shape function; $N_{b}$ is the number of particles within the support domain; $f(x_{i})$ is the physical quantity at the FEM node; and $\langle f(x)\rangle$ is the interpolated physical quantity at the SPH particle position.

Newly generated ghost particles require assignment based on the finite element nodes. The physical quantities at the FEM nodes can be dynamically transferred to the ghost particles at the interface, with the specific assignment procedure governed by the following rules:

(7) $$\left\{\begin{array}{c} x_{p}=x_{n}\\ m_{p}=m_{n}=\sum_{i=1}^{N_{e}}\frac{1}{N_{n}}\rho_{ei}V_{ei}\\ v_{p}=v_{n}\\ \sigma_{p}=\sigma_{n}=\frac{1}{N_{e}}\sum_{i=1}^{N_{e}}\sigma_{ei}=\frac{1}{N_{e}}\sum_{i=1}^{N_{e}}\sum_{j=1}^{N_{g}}w_{gj}\sigma_{gj}\\ c_{p}=c_{n} \end{array}\right.$$

where the subscript g is the Gaussian integration point; e is the element; n is the node; p is the particle; $w_{gi}$ is the integration weight; $N_{e}$ is the number of elements connected to the node; $N_{g}$ is the number of Gaussian points within the element; $\sigma_{ei}$ is the stress tensor; and $N_{n}$ is the number of nodes per element.

The smoothing length of the ghost particles is determined by the following equation:

(8) $$h_{p}=\frac{\alpha }{N_{e}}\sum_{i}^{N_{e}}r_{0i}{\left(\frac{\rho_{0i}}{\rho_{i}}\right)}^{\frac{1}{d}}$$

where $r_{0}$ is the initial element size; α is a constant; and d is the dimensionality.

To prevent non-physical penetration between SPH particles and the FEM interface, a contact force algorithm based on a potential function is employed. The contact force and contact potential are defined as follows:

(9) $$f_{c}=\int_{\Omega_{CBL}}N^{T}b_{c}dV$$

(10) $$\phi (x_{A})={\int_{\Omega_{CBL}}K(\frac{W(x_{A}-x_{B})}{W(\Delta p_{avg})})}^{n}dV$$

where N is the shape function matrix; b is the body force vector; and W is the space of weighting functions. The contact force is obtained through the gradient of the potential function as follows:

(11) $$b_{c}(x_{i})=\nabla \phi (x_{i})=\sum_{j}^{NCONT}\frac{m_{j}}{\rho_{j}}Kn\frac{W{\left(r_{ij}\right)}^{n-1}}{W{\left(\Delta p_{avg}\right)}^{n}}\nabla_{x_{i}}W(x_{i}-x_{j})$$

The contact force is ultimately incorporated as an external force term into the SPH momentum equation and the FEM equations, respectively:

(12) $$\frac{dv_{i}^{\alpha }}{dt}=\sum_{j=1}^{N}m_{j}(\frac{\sigma_{i}^{\alpha \beta }}{\rho_{i}^{2}}+\frac{\sigma_{j}^{\alpha \beta }}{\rho_{j}^{2}}-\Pi_{ij})\frac{\partial W_{ij}}{\partial x_{i}^{\beta }}-\frac{f_{c}(x_{i})}{m_{i}}$$

(13) $$M\ddot{u}+C\dot{u}+Ku=f_{c}(x_{i})$$

**Figure 7:**
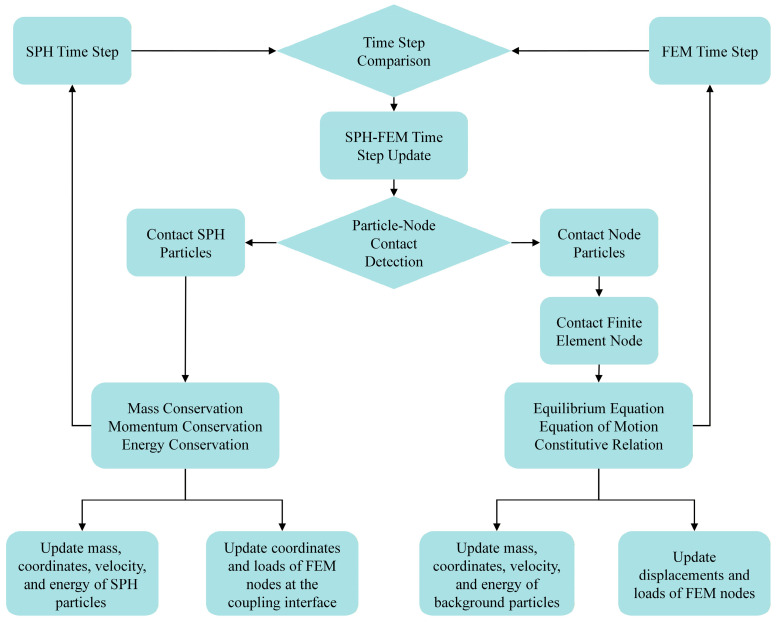
Flowchart of SPH-FEM contact algorithm.

The time step for the entire coupled system is governed by the more restrictive of the stability criteria from both the SPH and FEM methods:

(14) $$\Delta t_{cv}=\min(\frac{h_{i}}{c_{i}+0.6(a_{\Pi }c_{i}+\beta_{\Pi }\max(\phi_{ij}))})$$

(15) $$\Delta t_{FEM}=\frac{\lambda_{3}L_{\min}}{\sqrt{\frac{E(1-v)}{\rho (1+v)(1-2v)}}}$$

where $L_{\min}$ is the minimum element size; ρ is the density; v is Poisson’s ratio; $\lambda_{3}$ is a dimensionless constant; and E is the elastic modulus. The computational flowchart of the SPH-FEM contact algorithm is illustrated in Figure 7.

## 4. Numerical Simulation Model

This section establishes a mesoscopic numerical model of NEPE propellant based on the real meso-structure characterized by micro-CT in the experiment, and carries out numerical simulation of the drop-weight impact damage process using the SPH-FEM coupling algorithm introduced in Section 3.

### 4.1. Void and Crack Damage Model

In this work, a mesoscale numerical model of NEPE propellant is established, incorporating three damage evolution modes: microcracking, void collapse, and interfacial dewetting. In the model, the overall stress and strain are decomposed into deviatoric and volumetric parts, respectively, to formulate a microcrack-related deviatoric constitutive relationship and a microvoid-related volumetric constitutive relationship. The two parts are coupled through the Gurson yield criterion, and the relevant state variables are updated via the evolution equations for microcracks and microvoids.

The plastic deformation of the NEPE propellant is characterized by the classical Gurson model [37], in which the material yield surface involves the von Mises equivalent stress $\sigma^{e}$ and the hydrostatic pressure p as follows:

(16) $$F(\sigma^{e},p,f)={\left(\sigma^{e}/Y_{M}\right)}^{2}+2f\cosh(-3/2Y_{M})-f^{2}-1=0$$

To account for the material hardening behavior and strain rate sensitivity under dynamic loading, the yield strength $Y_{M}$ of the fully dense matrix material (f=0) can be expressed as:

(17) $$Y_{M}=(\sigma_{0}+h{\left({\bar{\varepsilon }}_{M}^{p}\right)}^{n})[1+C\cdot \ln(1+{\dot{\varepsilon }}^{*})]$$

where $\sigma_{0}$ is the initial yield stress under ${\dot{\varepsilon }}_{0}=10s^{-1}$ loading; ${\bar{\varepsilon }}_{M}^{p}$ is the equivalent plastic strain; ${\dot{\varepsilon }}^{*}=\dot{\varepsilon }/{\dot{\varepsilon }}_{0}$ is the equivalent plastic strain rate; h is the hardening modulus; and n and C are material parameters.

The macroscopic stress–strain response of the NEPE propellant is characterized by an elastoplastic damage constitutive model. The total strain rate ($\dot{\varepsilon }$) and stress rate ($\dot{\sigma }$) are decomposed into deviatoric $\left(\dot{e},\dot{s}\right)$ and volumetric $\left({\dot{\varepsilon }}_{v},-\dot{P}\right)$ components. The total deviatoric strain rate consists of three additive parts: a viscoelastic component (${\dot{e}}^{ve}$), a crack damage component $\left({\dot{e}}^{cr}\right)$, and a plastic component $\left({\dot{e}}^{p}\right)$, which respectively describe the effects of viscoelastic deformation, microcrack evolution, and plastic deformation of the matrix material on the mechanical properties of NEPE propellant. The viscoelastic deformation of the binder is described using a generalized Maxwell model, and the crack strain caused by microcrack opening and shear propagation is described by the SCRAM model. The deviatoric constitutive relation can be expressed as:

(18) $$\dot{s}=2GA\cdot (\dot{e}-{\dot{e}}^{p})-B\cdot (s+C)$$

where s is the deviatoric stress tensor; G is the shear modulus (Pa).

The material parameters *A*, *B*, and *C* are defined as follows:

(19) $$\left\{\begin{array}{l} A=\frac{1}{1+{\left(\bar{c}/a\right)}^{3}}\\ B=\frac{\alpha^{e}{\left(\bar{c}/a\right)}^{2}\dot{\bar{c}}/a}{1+{\left(\bar{c}/a\right)}^{3}}\\ C=\frac{\sum_{n=1}^{N}s^{\left(n\right)}/\tau^{\left(n\right)}}{3{\left(\bar{c}/a\right)}^{2}\dot{\bar{c}}/a} \end{array}\right.$$

where $\bar{c}$ is the mean microcrack size (mm); $\tau^{\left(n\right)}$ and $s^{\left(n\right)}$ denote the relaxation time (s) and deviatoric stress of the n-th Maxwell component (Pa), respectively.

The characteristic initial crack size a is calculated by the following expression:

(20) $$\left\{\begin{array}{c} a^{-3}=6G\beta \\ \beta =\frac{64\pi (1-v)N_{0}}{15(2-v)G} \end{array}\right.$$

where v and $N_{0}$ denote Poisson’s ratio and the initial crack number density (m^−3^), respectively.

Based on the Griffith energy release rate criterion for crack propagation, the microcrack evolution equation can be formulated as [38]:

(21) $$\bar{c}={\dot{c}}_{\max}[1-\frac{2\bar{\gamma }}{g_{dom}(\sigma ,\bar{c})}]$$

where ${\dot{c}}_{\max}$ is the maximum expansion velocity (m/s); $\bar{\gamma }$ is the specific surface energy of the material (J/m^2^). The term $g_{dom}(\sigma ,\bar{c})$ corresponds to the energy release rate associated with the dominant crack.

Due to the randomness of the orientation distribution of microcracks in the material, there exists a critical microcrack orientation corresponding to the maximum energy release rate. Microcracks along this orientation first propagate unstably under the minimum applied stress and are therefore defined as the main crack. The determination of the main crack orientation is related to the current stress state. The determination of the main crack orientation and the corresponding energy release rate can be found in References [38,39]. The damage degree of the material corresponding to microcrack propagation is defined as:

(22) $$d_{cr}={\bar{c}}^{3}/(a^{3}+{\bar{c}}^{3})$$

where $d_{cr}$ is the microcrack damage variable; a is the characteristic initial microcrack size (mm).

To describe the effect of irreversible damage caused by microvoid evolution on the volumetric deformation of NEPE propellant, a porosity-dependent equation of state [40] is adopted:

(23) $$p(\rho ,e,f)=(1-f)[\frac{\rho_{s}c_{0}^{2}\eta_{s}}{\left(1-s\eta_{s}\right)}(1-\frac{\Gamma_{s}\eta_{s}}{2})+\Gamma_{s}\rho_{s}e_{s}]$$

where f is the porosity; ρ is the density of the void-containing material (g/cm^3^); e is the specific internal energy (J/g). For the fully dense matrix material, $\rho_{s}=\rho /(1-f)$, $e_{s}=e$, and $\eta_{s}=1-\rho_{s_{0}}/\rho_{s}=-\varepsilon v$ correspond to its density, specific internal energy, and pressure, respectively; $\Gamma_{s}$ is the grisen coefficient; $c_{0}$ is the wave speed (m/s); s is a material-specific parameter. This equation is used to describe the change in pressure inside the propellant caused by void collapse under impact loading, and provides the mechanical basis for the calculation of void damage evolution.

Accounting for two distinct deformation mechanisms, porosity reduction $\left({\dot{f}}_{vc}\right)$ due to void collapse and porosity increase $\left({\dot{f}}_{vd}\right)$ induced by void deformation, the porosity evolution equation can be expressed as [41]:

(24) $$\dot{f}=(1-f){\dot{\varepsilon }}_{v}^{p}+fk_{w}\omega (\sigma )\frac{s_{ij}{\dot{e}}_{ij}^{p}}{\sigma_{e}}$$

where ${\dot{\varepsilon }}_{v}^{p}={\dot{\varepsilon }}_{kk}^{p}/3$ is the plastic volumetric strain rate (s^−1^); $k_{w}$ is a shear-dependent material parameter; ω is a stress state dependent variable with values ranging from 0<ω<1.

(25) $$\omega (\sigma )=1-{\left(\frac{27J_{3}}{2\sigma_{e}^{3}}\right)}^{2}$$

where $J_{3}$ is the third invariant of the stress tensor (MPa^3^).

The damage degree of an initial void is defined as the ratio of the void collapse displacement to its initial diameter, calculated by the following formula:

(26) $$d_{vo}=\frac{\delta_{vo}}{L_{vo}}$$

where $\delta_{vo}$ is defined as the void collapse displacement (mm), and $L_{vo}$ is the initial cavity diameter (mm).

### 4.2. Particle/Matrix Interface Damage Model

To simulate the interfacial dewetting damage between particles and the matrix in the mesostructure of the propellant, a bilinear CZM is incorporated into the FEM. The CZM characterizes the interfacial region as a bonding unit with specific strength. The bilinear CZM describes the interfacial behavior through three distinct stages: elastic deformation, damage evolution, and complete failure, which define the linear elastic relationship between interfacial traction and separation displacement before damage initiation. The corresponding traction-separation law governing this behavior is given by [42]:

(27) $$T=\left\{\begin{array}{l} T_{n}\\ T_{t}\\ T_{z} \end{array}\right\}=\left[\begin{array}{ccc} (1-D)K_{n} & 0 & 0\\ 0 & (1-D)K_{t} & 0\\ 0 & 0 & (1-D)K_{z} \end{array}\right]\left[\begin{array}{l} \varepsilon_{n}\\ \varepsilon_{t}\\ \varepsilon_{z} \end{array}\right]$$

where $T_{n}$, $T_{t}$, and $T_{z}$ are the interfacial stresses (MPa); $K_{n}$, $K_{t}$, and $K_{z}$ are the corresponding elastic stiffness coefficients (MPa·mm^−1^); $\varepsilon_{n}$, $\varepsilon_{t}$, and $\varepsilon_{z}$ are the interfacial strains.

D is the damage variable quantifying the extent of degradation, which is used to calculate the degradation degree of the interface bearing capacity after damage initiation, and describes the whole process of interface from damage initiation to complete failure defined as:

(28) $$D=\left\{\begin{array}{cc} 0 & \left(\delta \leq \delta^{0}\right)\\ \frac{\delta^{f}(\delta -\delta^{0})}{\delta (\delta^{f}-\delta^{0})} & \left(\delta >\delta^{0}\right) \end{array}\right.$$

where $\delta^{0}$ is the interfacial displacement (mm) at the onset of damage initiation; and $\delta^{f}$ is the interfacial displacement (mm) at complete failure. The condition D = 0 corresponds to the initial undamaged state of the interface, whereas D = 1 indicates that the interface has fully failed.

**Figure 8:**
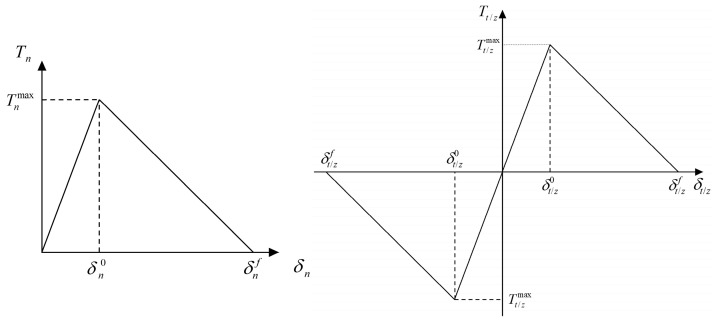
Bilinear CZM.

The constitutive relation of the interface is given by the following expression:

(29) $$\left\{\begin{array}{cc} \begin{array}{l} T=K\delta \\ T=(1+K/\tilde{K})T^{\max}-\tilde{K}\delta \\ T=0 \end{array} & \begin{array}{l} 0<\delta \leq \delta^{0}\\ \delta^{0}<\delta \leq \delta^{f}\\ \delta^{f}<\delta \end{array} \end{array}\right.$$

where $T^{\max}$ is the interfacial bonding strength (MPa); K and $\tilde{K}$ are the elastic modulus (MPa/mm) and softening modulus (MPa/mm) of the interface, respectively. The characteristic response curve of the model is illustrated in the accompanying Figure 8.

The bilinear CZM is defined by three parameters: initial stiffness, cohesive strength, and critical failure displacement. Its response has two stages: an elastic stage where traction increases linearly with separation, followed by damage initiation at point $\delta^{0}$ leading to stiffness degradation, until complete failure at point $\delta^{f}$.

Owing to the presence of numerous solid inclusions within the model, the internal shock wave propagates through particles of varying properties and sizes during the actual impact process, leading to a highly non-uniform stress distribution throughout the material domain. Consequently, a new stress-based criterion is adopted to determine the initiation of interfacial damage, whose governing expression is given by:

(30) $${\left(\frac{T_{n}}{T_{n}^{\max}}\right)}^{2}+{\left(\frac{T_{t}}{T_{t}^{\max}}\right)}^{2}+{\left(\frac{T_{z}}{T_{z}^{\max}}\right)}^{2}\geq 1$$

(31) $$\delta =\sqrt{\delta_{n}^{2}+\delta_{t}^{2}+\delta_{z}^{2}}$$

where $\delta_{n}$, $\delta_{t}$, and $\delta_{z}$ are the separation displacements in the normal and two tangential directions, respectively. Complete interfacial failure occurs when the criterion $\delta >\delta^{f}$ is satisfied.

### 4.3. Geometric Model Construction

**Figure 9:**
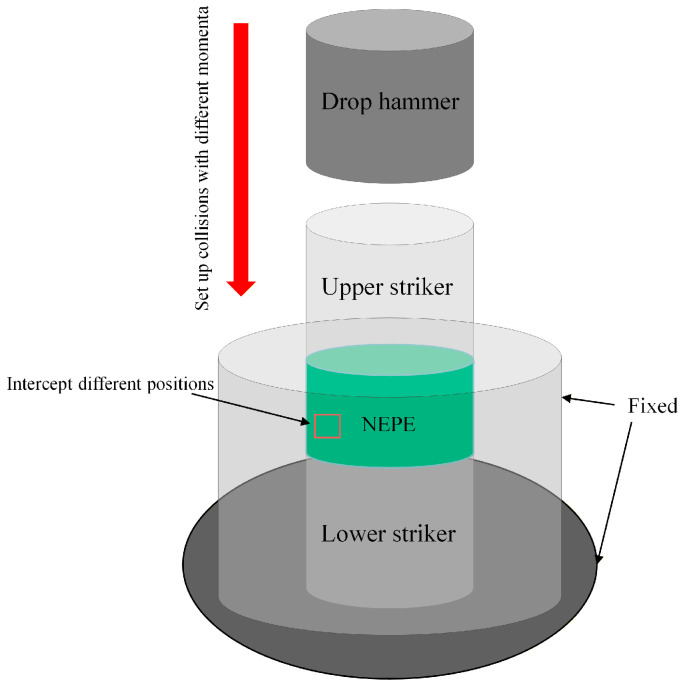
Schematic diagram of numerical calculation model.

Given the highly complex meso-structure of the propellant’s interior, the actual scenario is simplified to ensure computational convergence. Experimental results indicate that when the specimen thickness exceeds 3 mm, the characteristics and density of meso-damage in the near-surface region remain essentially consistent across different thicknesses. Therefore, the model is configured as an elastoplastic cylinder measuring Ø10 mm × 5 mm. Based on the actual formulation of the NEPE propellant, a random packing algorithm is employed to embed solid particles and voids within the model. Three types of solid particles are included: AP particles (50–100 μm), Al particles (20–30 μm), and HMX particles (20–50 μm). A schematic of the model is shown in Figure 9.

Based on extensive numerical calculations, the mechanical properties of the propellant matrix are determined as follows: an elastic modulus of 1.2 MPa, a Poisson’s ratio of 0.495, a yield strength of 2 MPa, and a fracture toughness of 0.9 MPa·m^1/2^. Given that the elastic modulus of the solid particles is significantly higher than that of the matrix, and considering that no particle fracture is observed in the experimental results, the particles are therefore modeled as elastic bodies with an elastic modulus of 32 MPa and a Poisson’s ratio of 0.1433. A bilinear CZM is implemented to simulate interfacial dewetting between the matrix and particles. As mentioned previously, the cohesive model requires three independent parameters: initial stiffness, cohesive strength, and failure displacement. Currently, there are no standardized values for the meso-interfacial mechanical parameters of propellants. Therefore, this study employs approximate interfacial properties to simulate the meso-damage in the solid propellant. Through multiple numerical experiments, a suitable set of interfacial parameters is calibrated, with the final values listed in Table 2.

The solid particles are discretized with 4-node linear tetrahedron elements, while most regions of the propellant matrix are modeled using 8-node cohesive elements. Only the local region around the initial voids is discretized by SPH particles. The cohesive elements, representing both the particle/matrix interface and matrix tearing, are defined as 4-node cohesive elements.

The NEPE propellant specimen is positioned between the upper and lower strikers, and the lateral surface of the specimen is in close contact with the fixed striker sleeve. The boundary conditions of the model are strictly set according to the actual drop-weight impact experiment: (1) The lower striker and the striker sleeve are set with full-degree-of-freedom fixed constraints; (2) The drop hammer only releases the translational degree of freedom along the impact axial direction, with the initial impact velocity consistent with the experiment applied; (3) The surface-surface contact boundary with a calibrated Coulomb friction model is set between the specimen and the upper/lower strikers, as well as between the specimen and the inner wall of the striker sleeve. Impact loading is applied by assigning varying magnitudes of initial velocity to the drop hammer.

### 4.4. Simulation Results Analysis

**Figure 10:**
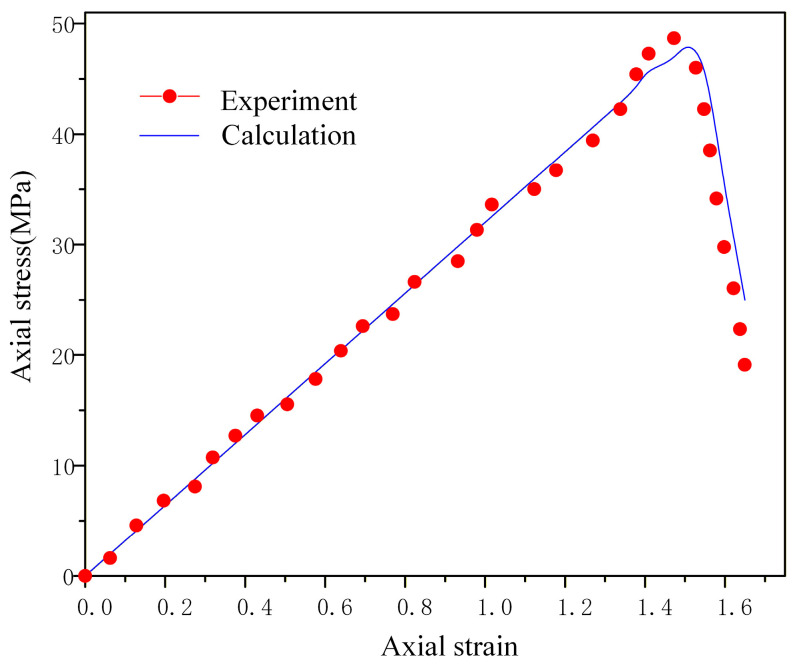
The stress–strain curve of the model.

The numerical results, as depicted in the stress–strain curve (Figure 10), exhibit strong agreement with the experimental data from the aforementioned drop-weight impact experiments. As meso-damage progressively accumulates, the macroscopic mechanical properties of the NEPE propellant are significantly compromised. Once the meso-damage reaches a critical threshold, observable macro-damage emerges in the propellant, accompanied by a marked increase in its sensitivity. This heightened sensitivity severely compromises the safety performance of solid rocket motors.

**Figure 11:**
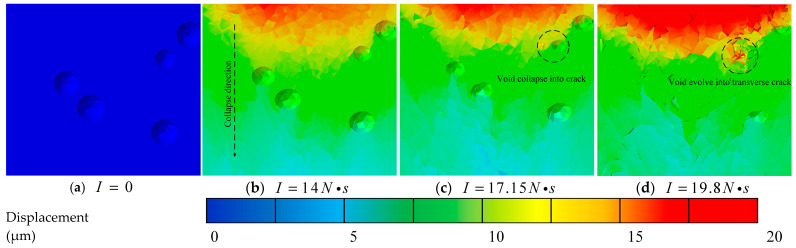
Void damage under different impulse loads.

Referencing the location characterized in the experimental analysis, a 200 μm cube is extracted from a depth of 1 mm beneath the center of the impacted model surface to observe its meso-damage evolution. Figure 11 presents cross-sectional views of the initial void damage in the propellant under different impact impulses.

**Figure 12:**
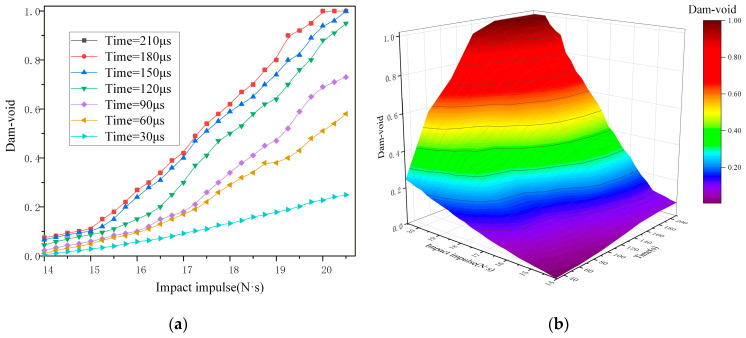
(**a**) Impact impulse-void damage curve and (**b**) 3D surface plot of Dam-void with impact impulse and time.

In Figure 11b, the direction of the black dashed line is the drop-weight impact direction. The dashed circle in Figure 11c,d represents the position of void collapse, and microcrack damage can be observed in the figure. Figure 12a illustrates the evolution curve of initial void damage, and Figure 12b shows the void damage 3D surface plot under the combined action of impact impulse and time. Analysis based on the damage cross-sections, curve and surface characteristics reveals the following regarding the influence of impact impulse: under low impact impulses, the initial voids exhibit negligible collapse due to the viscoelastic nature of the NEPE propellant. When the impulse exceeds 14 N·s, the initial voids begin to collapse, primarily along the tangential direction relative to the propagation path of the mechanical wave. As the impact impulse further increases, the void collapse intensifies and progressively evolves into micro-cracks. When the impact impulse exceeds 19.8 N·s, the initial voids collapse into transverse cracks, marking the transition from void-dominated damage to crack-dominated damage.

Temporally, due to the viscoelastic properties of the NEPE propellant, the external impact does not act directly on the voids. The resulting mechanical wave reaches its peak intensity at the void locations around 30 μs, after which void collapse intensifies and the void diameter decreases. The curve indicates a plateau in void damage progression between 180 μs and 210 μs, with only minor changes observed from 150 μs to180 μs. This suggests that the primary stage of void collapse occurs between 30 μs and 150 μs. As the mechanical wave intensity diminishes, the damage evolution decelerates progressively from 150 μs to 180 μs until it effectively ceases. With increasing impact impulse, the primary stage of void collapse damage shifts to earlier times. When the impact impulse exceeds 17.15 N·s, void damage is concentrated within 30 μs to 120 μs, with the main damage stage occurring between 30 μs and 90 μs.

**Figure 13:**
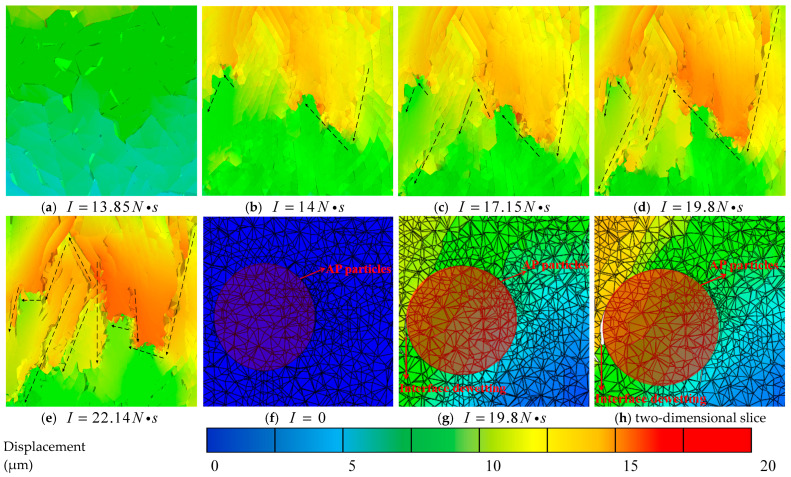
(**a**–**e**) Damage of propellant cracks and (**f**–**h**) dewetting damage of particle/matrix interface under different impulse impacts.

For the same 200 μm cube, the evolution of mesoscopic crack damage is observed. Figure 13 presents cross-sectional views of crack damage and particle/matrix interfacial dewetting damage in the propellant under different impact impulses.

**Figure 14:**
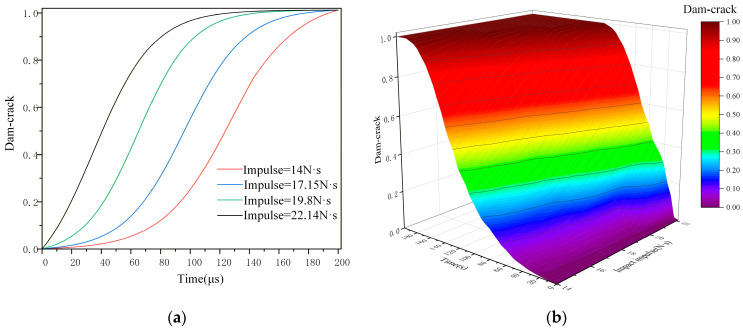
(**a**) Micro-crack damage evolution curve and (**b**) 3D surface plot of Dam-crack with time and impact impulse.

The black dashed lines in Figure 13b–e indicate the locations of the cracks, and the directions of the arrows represent the extension directions of the cracks. Figure 14a shows the damage evolution curve of a single microcrack, and Figure 14b shows the damage 3D surface plot of microcrack under the combined action of time and impact impulse. As revealed by the numerical results, when the impact impulse reaches 13.85 N·s, initial micro-cracks emerge sparsely within the matrix. When the impact impulse exceeds 14 N·s, the micro-cracks begin to propagate and evolve. Due to the reflection and superposition of mechanical waves at the particle/matrix interfaces, newly formed micro-cracks are predominantly localized near these interfaces and around existing voids. When the impact impulse exceeds 17.15 N·s, the micro-cracks undergo further propagation and extension, exhibiting limited coalescence. Concurrently, damage evolution via void collapse contributes to an overall increase in crack density throughout the model. When the impact impulse exceeds 19.8 N·s, the collapse of initial voids into transverse cracks lead to a marked increase in crack density. The micro-cracks progressively widen and deepen along their initial propagation paths, eventually coalescing to form dominant macro-cracks. At the model scale, this coalescence results in a net reduction in the total number of individual cracks, while simultaneously generating new micro-cracks around the primary macro-crack tips. Concurrently, dewetting initiates at the interfaces between particles and the binder matrix. When the impact impulse exceeds 22.14 N·s, matrix tearing and interfacial dewetting intensifies progressively, evolving toward crack formation and merging with micro-cracks to form large-scale macro-cracks. At the macroscopic level, significant matrix tearing damage becomes clearly observable.

Temporally, micro-crack damage primarily occurs within the 0–200 μs timeframe following impact. While an increase in impact impulse leads to larger micro-crack dimensions and a corresponding prolongation of their evolution duration, the numerical results indicate that the primary phase of micro-crack evolution shifts toward earlier times with increasing impact impulse. When the impact impulse is 14 N·s, micro-crack damage evolution is essentially completed between approximately 100 μs and 180 μs; When the impact impulse is 22.14 N·s, micro-crack damage evolution is completed between 20 μs and 60 μs. Consequently, micro-crack dimensions are not the governing factor for the damage evolution duration; rather, the increase in impact impulse accelerates the micro-crack damage evolution rate.

### 4.5. Mesoscopic Damage Model and Function Establishment

**Figure 15:**
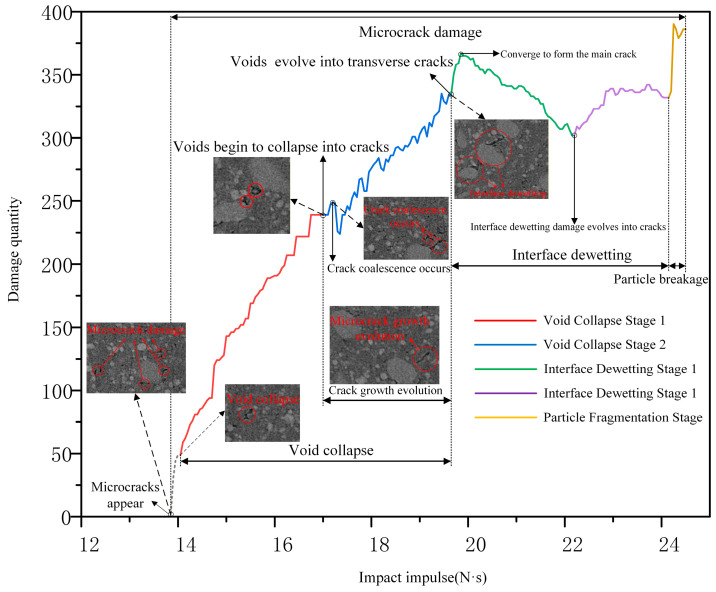
Mesoscopic damage model of NEPE propellant.

The types and quantities of internal damage within the extracted 200 μm surface unit are statistically analyzed, and the resulting meso-damage model curve for the NEPE propellant is presented in Figure 15.

The meso-damage modes incorporated in the developed damage model primarily comprise micro-cracking, void collapse, matrix tearing, and particle fragmentation. The model curve reveals that micro-crack damage persists throughout the entire meso-damage evolution process of the NEPE propellant. When the external impulse is below 13.85 N·s, no meso-damage manifestation is observed within the propellant, its overall physicochemical properties remain unaffected, and the solid propellant remains in a safe state. Once the external impulse exceeds 13.85 N·s, the meso-structure enters the micro-crack damage stage, signifying the initiation of the propellant’s meso-damage phase. When the impact impulse reaches 14 N·s, the meso-structure enters the void collapse stage, which is subdivided into two distinct phases. During the first phase, the voids undergo primarily longitudinal collapse due to the action of mechanical waves, leading to an increase in the total meso-damage quantity. When the external impulse reaches 17.15 N·s, the second phase is initiated. During this phase, a portion of the voids collapse into transverse cracks, while some micro-cracks coalesce due to propagation, resulting in a net reduction in the overall meso-damage quantity. When the external impulse reaches 19.8 N·s, all initial voids are fully transformed into transverse cracks due to mechanical wave action, and the meso-structure enters the particle/matrix interfacial dewetting stage. This stage is further subdivided into two distinct sub-stages. In the first sub-stage, interfacial dewetting initiates between particles and the matrix, while propagating micro-cracks coalesce into dominant macro-cracks. The formation of these macro-cracks leads to a reduction in the overall meso-damage quantity; When the external impact impulse reaches 22.14 N·s, the system enters the second sub-stage of interfacial dewetting. The intensified dewetting evolves into crack-dominated damage, resulting in a marked increase in the overall meso-damage quantity. The dominant macro-cracks cause substantial damage to the matrix, manifesting as macroscopic matrix tearing. At this point, the mechanical properties of the NEPE propellant undergo significant degradation, and its sensitivity progressively increases with accumulating damage. This condition has severely compromised the safety of the solid rocket motor, necessitating immediate inspection and maintenance to prevent potential accidents. When the external impact impulse exceeds 24.25 N·s, the solid particles within the propellant experience intense viscous shear, resulting in transgranular fracture and fragmentation. This process is accompanied by the formation of localized hot spots, ultimately leading to the ignition of the NEPE propellant.

The overall damage degree of the propellant is defined as the ratio of the meso-damage volume to the total model volume:

(32) $$d_{tot}=\frac{V_{meso\text{-}damage}}{V_{model}}$$

where $d_{tot}$ represents the overall damage degree; $V_{meso\text{-}damage}$ denotes the meso-damage volume (mm^3^); and $V_{model}$ is the model volume (mm^3^).

**Figure 16:**
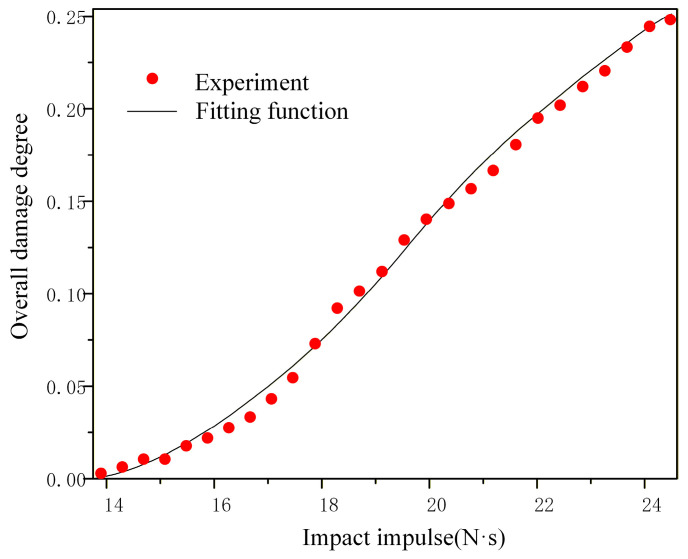
The curve of overall damage degree impact impulse.

The relationship between the overall damage degree and the impact impulse, obtained by fitting the numerical results, can be effectively described by a Boltzmann function, $R^{2}=0.99984$. The function is expressed as follows:

(33) $$d_{tot}=a+\frac{b-a}{1+e^{(I-I_{0})/c}}$$

where a = 0.288; b = −0.026; $I_{0}$ = 19.78; c = 2.429; I is the impact intensity (N·s), 13.85 N·s ≤ I < 24.25 N·s; a comparison between the damage function and the experimental results is presented in Figure 16.

As can be seen from the figure, the experimental results show strong agreement with the fitted function curve. Thus, the damage function enables rapid and reliable assessment of the damage degree in NEPE propellant subjected to impact impulses ranging from 13.85 N·s to 24.25 N·s. The above numerical simulation results are in good agreement with the experimental phenomena, reveal the dynamic evolution mechanism of each mesoscopic damage mode under increasing impact impulse, and verify the critical impulse threshold of each damage stage obtained from the experiment.

## 5. Conclusions

To address the research gap that the meso-damage mechanisms of NEPE propellant under drop-weight impact remain unclear, and to meet the requirements of impact safety risk assessment of solid rocket motors under accidental impact, this study systematically investigates the mesoscopic damage evolution law and quantitative damage model of NEPE propellant under drop-weight impact by combining drop-weight impact experiment, micro-CT, and SPH-FEM coupling numerical simulation. The main conclusions are summarized as follows.

(1) Based on 240 groups of drop-weight impact experiments with 10 specimen thickness gradients and 8 drop height gradients, the quantitative relationship between the mesoscopic damage of the NEPE propellant in this formulation and the impact impulse is clarified, and the key influencing factors and evolution paths of the mesoscopic damage and ignition response are obtained. The ignition threshold is jointly determined by the specimens’ thickness and the impact impulse. When the specimens thickness exceeds 5 mm, the impact ignition impulse stabilizes at 24.25 N·s, and the meso-damage characteristics in the subsurface region remain consistent across different thicknesses. Based on the micro-CT reconstruction images and porosity calculation, the damage forms of the unignited specimens under impacts with different impulses are mainly void collapse, matrix tearing and particle/matrix interface debonding. When the impact impulse is less than 24.25 N·s, no particle fragmentation and transgranular fracture phenomena are observed in the micro-CT characterization results.
(2) Based on the meso-morphological features characterized by micro-CT, an elastoplastic cylindrical model containing randomly distributed AP, Al, and HMX particles along with voids is constructed. Combined with the deviatoric constitutive model, the Gurson plastic damage model, and the bilinear CZM, the SPH-FEM coupling algorithm is adopted to achieve the accurate numerical simulation of the impact damage process. The numerical results demonstrate that the stress–strain curve of the model is in good agreement with the experimental results. Meanwhile, the characteristics and evolution patterns of mesoscopic damage are identified verifying the effectiveness of this numerical model in simulating the impact damage of NEPE propellant.
(3) A quantitative “impact impulse-mesoscopic damage” model is developed by integrating experimental and numerical simulation results. This model categorizes the impact response of NEPE propellant into five distinct stages, i.e., “undamaged stage-microcrack stage-void collapse stage-interfacial debonding stage-particle fragmentation”. It also quantifies the damage modes, evolution rates, and critical impulse thresholds associated with each stage. The damage function is obtained by fitting the computational results using a Boltzmann function, enabling quantitative description of the damage degree and demonstrating strong agreement with experimental data. The defined critical impact impulse for damage, the established quantitative damage degree function, and the “experiment-observation-simulation” technical method are oriented to the accidental drop or collision impact scenario during the production, transportation, and storage of solid rocket motors. Within the corresponding impact impulse range of 13.85 N·s to 24.25 N·s, the conclusions can be applied to evaluate the impact safety risks of NEPE propellant with conventional engineering charge sizes, offering references for structural optimization of propellant charges and safety design of solid rocket motors.

For subsequent research, the Split Hopkinson Pressure Bar method will be adopted to carry out systematic investigation on the mesoscopic damage characteristics of NEPE propellant under high-strain rates, to compare the differences in damage evolution mechanisms under different strain rate conditions, and establish a full-range strain rate damage model of NEPE propellant. Meanwhile, a thermal-mechanical fully coupled mesoscopic model will be established to carry out quantitative research on the impact-induced ignition mechanism of NEPE propellant, based on the damage evolution law revealed in this work, so as to further improve the impact safety assessment system of solid rocket motors.

## Figures and Tables

**Table 1:** Impact sensitivity experiment parameters.

Specimens Thickness/(mm)	Drop-Weight/(cm)	Number of Groups
2.5, 3, 3.5, 4, 4.5, 5, 5.5, 6, 6.5, 7	15, 20, 25, 30, 35, 40, 45, 50	240

**Table 2:** Interface parameter.

Interface Type	Initial Stiffness (MPa·mm^−1^)	Bond Strength (MPa)	Failure Displacement (mm)
Particle/matrix interface	100	0.5	0.02
Weak interface layer	80	0.4	0.015
Matrix element interface	80	0.6	0.035

## Data Availability

The data presented in this study are available on request from the corresponding author, as the data originate from an ongoing research project that has not yet been concluded.
